# P-638. Epidemiological Profile of Respiratory Syncytial Virus (RSV) in 187 Adult Patients at Mexico Hospital in Costa Rica (2022-2024)

**DOI:** 10.1093/ofid/ofaf695.851

**Published:** 2026-01-11

**Authors:** Jose A Castro Cordero, Juan Villalobos Vindas

**Affiliations:** Caja Costarricense de Seguro Social, Uruca, San Jose, Costa Rica; Caja Costarricense de Seguro Social, Uruca, San Jose, Costa Rica

## Abstract

**Background:**

Respiratory Syncytial Virus (RSV) is recognized as a significant cause of morbidity and mortality in adults, especially in vulnerable populations. However, limited data exists regarding its behavior in adult populations in Latin America. To characterize the clinical-epidemiological profile of adult patients with RSV infection in a tertiary hospital in Costa Rica.

**Figure:**
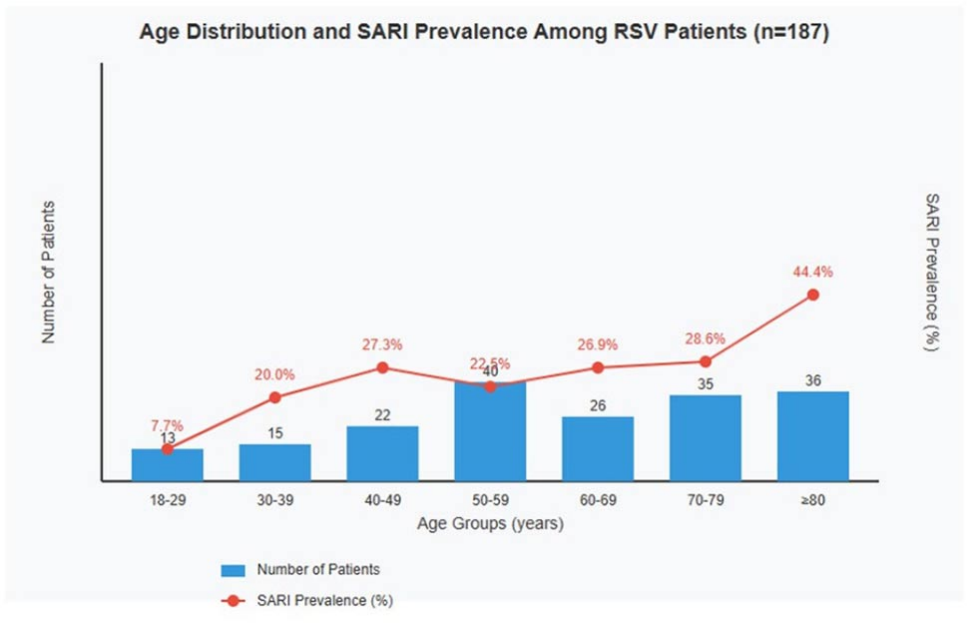
Age Distribution and SARI Prevalence Age Distribution and SARI Prevalence Among RSV Patients (n=187)

**Figure:**
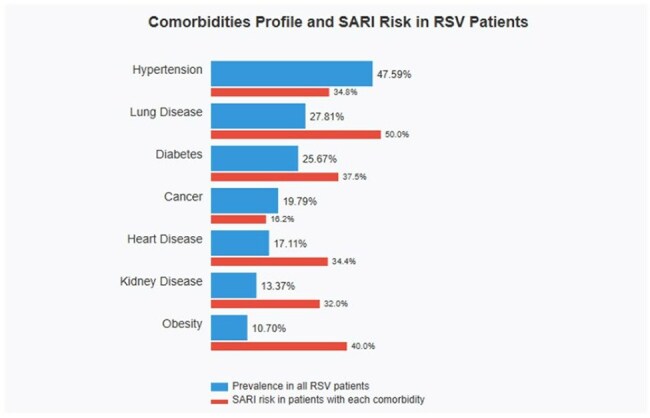
Comorbidities Profile Comorbidities Profile and SARI RIsk in RSV Patients

**Methods:**

Retrospective study of 187 adult patients with PCR-confirmed RSV diagnosis at Mexico Hospital between 2022-2024. Sociodemographic variables, comorbidities, clinical manifestations, and temporality were analyzed.

**Figure:**
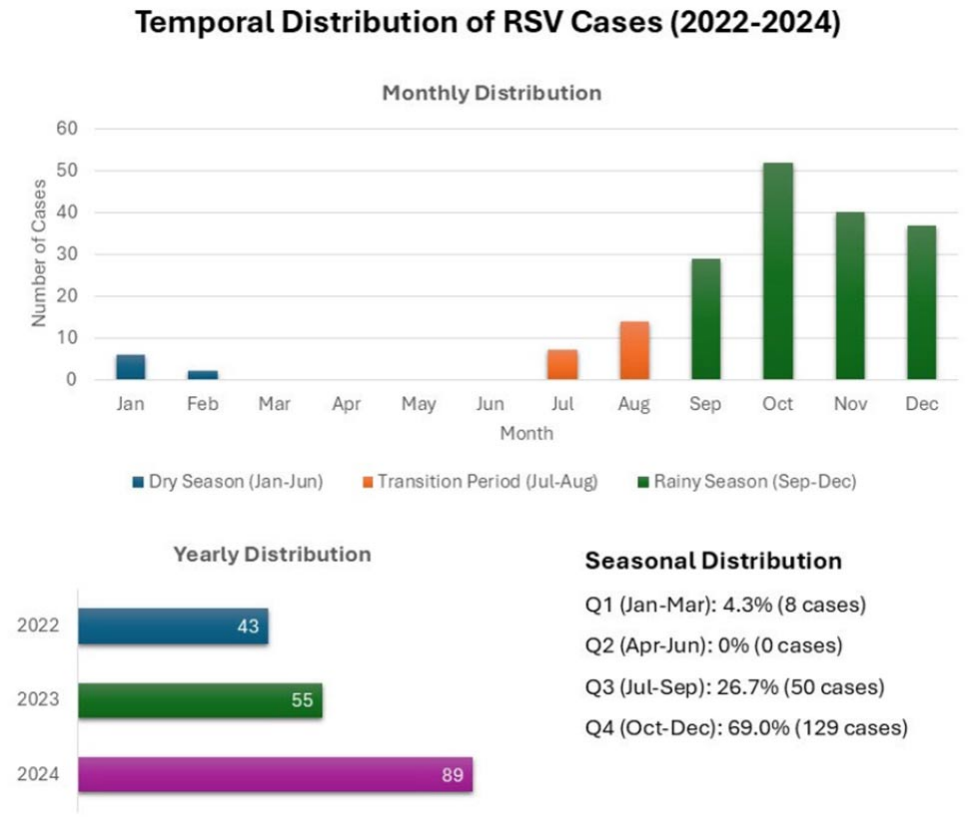
Temporal Distribution Temporal Distribution of RSV Cases (2022-2024)

**Results:**

Mean age was 60.85 years (range 18-104), with female predominance (61.5%). 51.9% of cases occurred in patients over 60 years. Most frequent comorbidities were hypertension (47.6%), lung disease (27.8%), diabetes (25.7%), and cancer (19.8%), with 83.4% of patients presenting at least one comorbidity. Predominant symptoms were cough (73.8%), dyspnea (48.1%), rhinorrhea (28.3%), and fever (24.6%). 27.8% developed Severe Acute Respiratory Infection (SARI), reaching 44.4% in patients ≥80 years. A progressive increase in cases was observed (22.99% in 2022, 29.41% in 2023, and 47.59% in 2024), with marked seasonality: 69.0% of cases in October-December, 26.7% in July-September, and complete absence in April-June.

**Conclusion:**

RSV infection in adults predominantly affects older adults with comorbidities, with one-fourth developing SARI. Clear seasonality can guide preventive strategies and healthcare resource planning. The growing trend of cases suggests the need to strengthen surveillance systems and consider specific preventive measures, including future RSV vaccination in high-risk populations.

**Disclosures:**

All Authors: No reported disclosures

